# Developing a Smartwatch-Based Healthcare Application: Notes to Consider

**DOI:** 10.3390/s23156652

**Published:** 2023-07-25

**Authors:** Ramin Ramezani, Minh Cao, Arjun Earthperson, Arash Naeim

**Affiliations:** 1 Center for Smart Health, University of California, Los Angeles, CA 90095, USA; mcao21@ucla.edu (M.C.); anaeim@mednet.ucla.edu (A.N.); 2 Probabilistic Risk Assessment Group, North Carolina State University, Raleigh, NC 27613, USA; aarjun@ncsu.edu

**Keywords:** remote patient monitoring, smartwatch application, smartwatch app, Android, Wear OS, telehealth, telemedicine

## Abstract

Wearable devices and fitness trackers have gained popularity in healthcare and telemedicine as tools to reduce hospitalization costs, improve personalized health management, and monitor patients in remote areas. Smartwatches, particularly, offer continuous monitoring capabilities through step counting, heart rate tracking, and activity monitoring. However, despite being recognized as an emerging technology, the adoption of smartwatches in patient monitoring systems is still at an early stage, with limited studies delving beyond their feasibility. Developing healthcare applications for smartwatches faces challenges such as short battery life, wearable comfort, patient compliance, termination of non-native applications, user interaction difficulties, small touch screens, personalized sensor configuration, and connectivity with other devices. This paper presents a case study on designing an Android smartwatch application for remote monitoring of geriatric patients. It highlights obstacles encountered during app development and offers insights into design decisions and implementation details. The aim is to assist programmers in developing more efficient healthcare applications for wearable systems.

## 1. Introduction

Over the past decade, the surge in demand for personalized health and home care has led to the widespread adoption of wearable fitness tracking technology, exemplified by devices such as FitBit or Misfit [1]. These advanced sensor-equipped devices enable the monitoring of various health parameters, including activity levels, sleep patterns, heart rate, and estimation of energy expenditure [2,3,4,5].

In recent years, there has been a notable shift in the wearables market, with users seeking more versatile functionality beyond traditional fitness trackers. Smartwatches have emerged as a preferred choice due to their increasing embedded capabilities, fulfilling the desires of individuals who seek both fitness tracking and general watch features [6,7]. Importantly, smartwatches not only facilitate fitness tracking but also enable user interaction, phone calls, and seamless integration with widely adopted cloud services such as Amazon Web Services AWS and Microsoft Azure [8,9]. Notably, these cloud services have established partnerships with renowned healthcare providers [10,11], paving the way for the integration of fitness data from wearable devices with electronic health records (EHR).

As wearable fitness technology becomes increasingly prevalent among the public, healthcare professionals are actively exploring ways to integrate the collected fitness data from these devices with electronic health records. This integration holds particular significance for frail geriatric patients living without supervision. These patients face the risk of experiencing a decline in overall well-being, increased dependency, and a higher susceptibility to fall-related injuries due to deteriorating motor performance. Leveraging noninvasive health tracking devices, especially during the postsurgery recovery period, offers substantial benefits and is well received by patients for extended monitoring purposes [12,13,14,15].

The integration of smartwatches into patient monitoring systems is currently in its early stages, and several challenges need to be addressed. A successful integration of these technologies into healthcare relies on the active involvement and acceptance of patients, emphasizing the importance of their digital literacy [16,17]. This paper aims to elucidate the implementation challenges encountered by developers when constructing healthcare applications on smartwatches. We thoroughly examine these challenges and present potential solutions, wherever feasible. The improvement of efficiency and reliability in smartwatch healthcare applications holds the potential to positively impact patient adherence to utilizing such technologies. By providing user-friendly and dependable applications, patients are more likely to engage effectively and adhere to the use of these technologies.

This paper is structured as follows: Section 2 explores the potential of commercially available smartwatches for developing healthcare applications. In Section 3, the guidelines for ensuring patient data privacy, availability, and security are outlined, emphasizing the necessary compliance measures in healthcare product development. Section 4 provides detailed information on the distribution, monitoring, and remote control of our application. It explains how the smartwatches’ sensors collect user data while conserving battery life. Section 5 covers important configuration and setting details that can enhance the performance of monitoring applications. Lastly, Section 6 elucidates several constraints associated with applications developed on smartwatch platforms.

## 2. The Potential Role of Smartwatches in Healthcare Applications

The proliferation of consumer electronic devices equipped with embedded computing and connectivity capabilities has facilitated the development of the Internet of Things (IoT), enabling a network of interconnected devices to exchange data with remote cloud services. Capitalizing on this advancement, healthcare researchers have seized the opportunity to harness personal health mobile devices, including FitBit and smartwatches, for the purpose of remote patient monitoring. This innovative approach presents a cost-effective solution whereby patients can receive clinical monitoring while maintaining their presence outside the confines of traditional healthcare settings [7].

Smartwatches have emerged as a key component of remote health monitoring systems, owing to several advantages.

**Figure 1:**
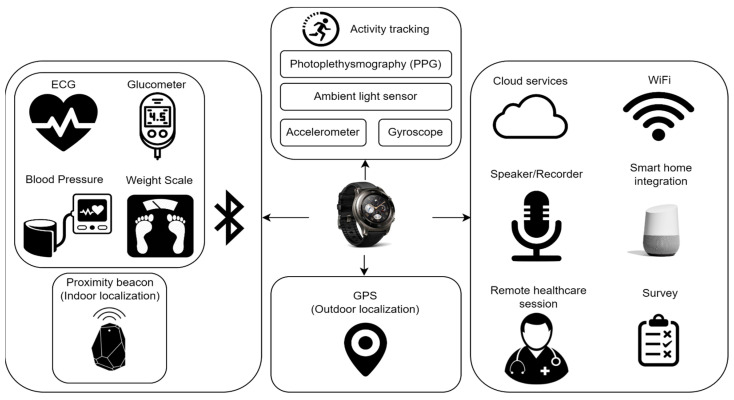
Potential usage of smartwatch sensors and antennas.

Figure 1 presents a comprehensive collection of nonintrusive wearable sensors, seamlessly integrated into a smartwatch application for remote monitoring purposes. These commercially available sensors offer diverse health monitoring capabilities, emphasizing their widespread accessibility and potential for adoption in the commercial market. However, it should be noted that Figure 1 only represents a portion of the various sensors utilized in remote patient monitoring (RPM) systems. Recent advancements in sensor technology, such as flexible sensors and electronic skins, are actively investigated for potential integration into healthcare and medical monitoring equipment [18].

Contemporary smartwatches are equipped with embedded sensors that enhance their health monitoring functionalities, including step counting, energy expenditure estimation, heart-rate monitoring, activity tracking, and sleep analysis. Additionally, smartwatches offer supplementary features that enable healthcare developers to provide feedback and engage in interactive tasks through touchscreens, speakers, and audio recorders [19,20].

In summary, Figure 1 provides an illustrative representation of commercially available wearable sensors, acknowledging ongoing advancements in sensor technology and the added capabilities of embedded sensors within smartwatches. These innovations have the potential to revolutionize remote patient monitoring applications, leading to improved accessibility and precision in healthcare. Moreover, the connectivity of smartwatches is enhanced through Bluetooth technology, facilitating connections with various Bluetooth-compatible sensor devices, expanding the scope of their applications [21]. A few examples would be thermometers, sphygmomanometers (blood pressure monitors), oximeters (blood oxygen level monitors), and electrocardiograms (ECG), harnessed with either Bluetooth Generic Attribute Profile protocol (GATT) [22] or the custom Software Development Kit (SDK) provided by the devices, with an example that can be found in [23]. Note that the chest strap Bluetooth ECG device shown in the figure can be utilized for continuous monitoring. While some smartwatches have ECG monitoring capabilities, it is important to note that as of the time of writing this paper, these smartwatches only support spot-check ECG measurements at 30 s intervals. Moreover, users are required to actively hold the electrodes during the measurement process. In contrast, the chest strap device enables passive ECG data collection, providing the advantage of continuous monitoring without the need for active user involvement. Moreover, Wi-Fi connectivity enables smartwatches to serve as local hubs, facilitating real-time, continuous monitoring by transferring data from the watch and its locally connected devices to remote cloud services.

Among all the platforms available for smartwatches, we identified WearOS, the Android-based operating system developed by Google, as the most suitable platform for healthcare researchers. The WearOS platform inherits the flexibility and extensive community support from the Android open-source system, providing a favorable environment for application development. Additionally, due to the widespread popularity of Android as a mobile platform, Wear OS is highly accessible. Major cloud service providers, including Amazon Web Services (AWS), Azure, and Google Cloud Platform (GCP), offer Android SDKs that seamlessly integrate with Wear OS, enabling crucial functionalities such as authentication, file uploading, and remote configuration for remote health monitoring.

It is worth noting that the Wear OS platform enforces specific structures and policy requirements during application development to uphold standard quality. Notably, there are limitations on network and CPU-intensive tasks for applications running in the background when the user is not actively engaging with them. This restriction presents challenges for long-running background health monitoring applications seeking to gather continuous data. To address this issue, we introduce a comprehensive application framework supplemented with code examples. This framework aims to aid healthcare developers in ensuring the proper functionality of their applications while adhering to the policy requirements of the Wear OS operating system [24,25].

## 3. Cloud and HIPAA Compliancy

Professionals involved in the design and development of healthcare infrastructures, particularly in remote patient monitoring and Internet of Things (IoT) systems, play a vital role in ensuring the availability, security, and privacy of patient data. This responsibility entails strict adherence to the guidelines outlined in the Health Insurance Probability and Accountability Act (HIPAA) during healthcare product development. These guidelines encompass data security and privacy standards for medical records and must be integral to the development of IoT platforms [26,27]. Key aspects of ensuring HIPAA compliance include the following:

1. Authorization access: Access to medical data, electronically and physically, is restricted to authorized individuals or relevant patients.
2. Secure encryption: Medical data, whether at rest or in transit, must be encrypted.
3. Audit report: A comprehensive logging system records all data-accessing activities, documenting the specific user, the data section accessed, and the corresponding timestamps.
4. Backup policies: To safeguard against disasters or failures, data in storage systems are replicated, thereby preserving data integrity.

In our system, we utilize the services of premier cloud service providers like AWS and Azure, which incorporate these facets in their service configurations. These providers also offer Software Development Kits (SDKs) that aid in establishing secure connections, thereby enhancing data security from patients to cloud servers.

To ensure HIPAA regulations, we implemented the following stipulations in our smartwatches:

1. Information revealing patients’ identity is not archived on the smartwatches; instead, patient associations are drawn from unique IDs on a secure server.
2. Data accumulated by the watches are encrypted and housed in the application’s exclusive folder. OS-provided storage sandboxes ensure that these data are not leaked to other apps.
3. The smartwatch application records all executed tasks for security and debugging.
4. Data transmission from the watches to the cloud storage employs the HTTPS protocol, with every transaction logged for audit.
5. Multifactor authentication is required for read access to cloud storage.
6. Access to cloud server requires Virtual Private Network (VPN) access and robust password protections.
7. In the event of smartwatch compromise, the cloud monitoring service can trigger a factory reset command via an associated administrative application, thereby erasing all user data, inclusive of our application. Additionally, the IoT-Hub can invalidate the authentication of compromised smartwatches.

## 4. System Overview

**Figure 2:**
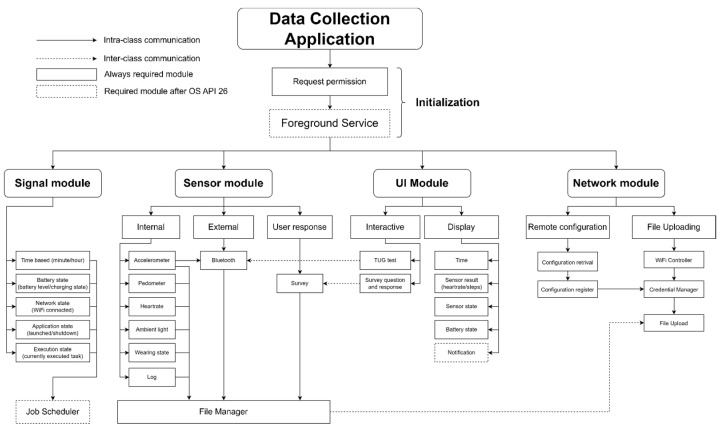
Overview of the system architecture of our proposed healthcare monitoring application.

**Figure 3:**
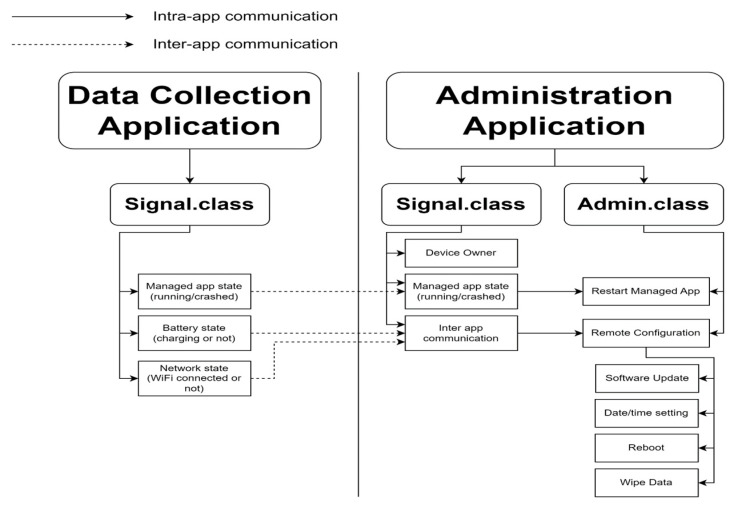
The data collection application communicates its status such as application state, battery state, and network state to the administration application to determine the optimal timing for implementing remote configuration and software updates.

The proposed general structure of the healthcare application for smartwatches on Wear OS, as depicted in Figure 2, comprises the following components:

1. Data collection application initialization: This stage ensures compliance with Android policy requirements for the data collection application to run in the background continuously. It involves requesting permissions for various sensors, network capabilities, and storage. Additionally, the initialization stage consolidates all long-running tasks into a foreground service to circumvent interference from the OS doze mode, which is discussed in the subsequent section.
2. Signal class: This component facilitates the coordination of actions among various components of the data collection application. As healthcare monitoring tasks are primarily asynchronous background processes involving sensor data collection and storage, a reliable signal system is crucial for timely message delivery.
3. Sensor class and data collection: Serving as the core of the data collection application, this class gathers sensor data from the smartwatch and any connected Bluetooth sensors. Data collection occurs passively in the background and actively during user interaction events. To ensure secure storage, the collected data are encrypted using the Advanced Encryption Standard (AES) algorithm. Given that the sensor class operates continuously, battery conservation measures must be implemented without compromising data quality.
4. User interface (UI) class: This class provides health monitoring status feedback to patients and handles user interaction events. Notably, in smartwatch UIs, any UI changes must be executed on the UI thread (main thread). Consequently, long-running tasks should be delegated to the background thread to prevent UI freezing.
5. Network and monitoring class: Responsible for communication with remote cloud services, this class encompasses authentication, data uploading, remote configuration synchronization, and adaptive online user interaction events. Detailed information regarding these network tasks is covered in section VII.B, while strategies to overcome unreliable connections are discussed in section VIII.E.
6. Data collection application deployment through administration application: The administration application, developed separately from the data collection application, possesses elevated permissions and monitors the behavior of the data collection application. The interaction between the administration application and the data collection application is illustrated in Figure 3. The data collection application communicates its status such as application state, battery state, and network state to the administration application, which utilizes this acquired data to assess the optimal timing for implementing remote configuration and software updates. For instance, the administration application waits until the Wi-Fi connectivity is accessible and the device is actively charging to initiate software updates, thereby ensuring efficient and uninterrupted operations. Additionally, the administration application performs system updates. By maintaining a separate administration application, privileges such as software updates, watch configuration, and data wiping actions can be limited exclusively to the administration application.

### 4.1. Data Collection Application Initialization

The Wear OS platform enforces runtime policies to ensure user control over application behavior and preserve device battery life. Two critical policies are (1) permission requests for utilizing device resources and (2) minimizing background activity during doze mode, which are elaborated upon in the subsequent paragraphs. Noncompliance with these policies severely impacts the functionality of healthcare applications.

**Figure A1:**
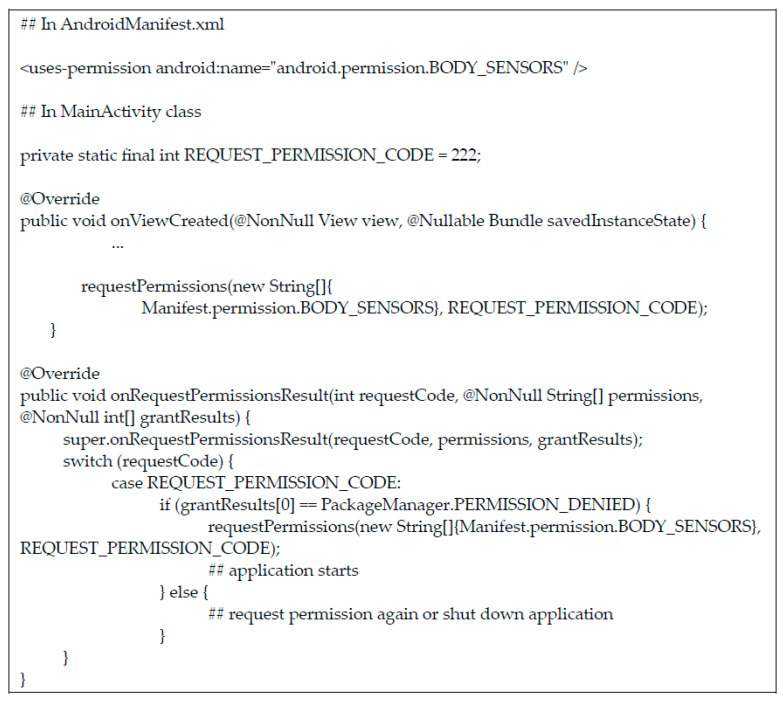
Example Android studio java code to request and check for permission.

All Wear OS applications must explicitly request user permission to access hardware components and sensitive data. Permission requests can be made either upon application launch or just before performing actions that require specific permissions (e.g., requesting permission for Bluetooth sensor usage prior to scanning for nearby Bluetooth proximity sensors). Once a permission request is submitted, users receive a prompt to grant or deny permission for the requested action. Generally, users are prompted only once for permission grants, unless they choose the “one-time permission” option. Table 1 provides an overview of actions that require specific permissions, along with their corresponding permission names. A sample code demonstrating permission requests is presented in Appendix A Figure A1.

**Figure 4:**
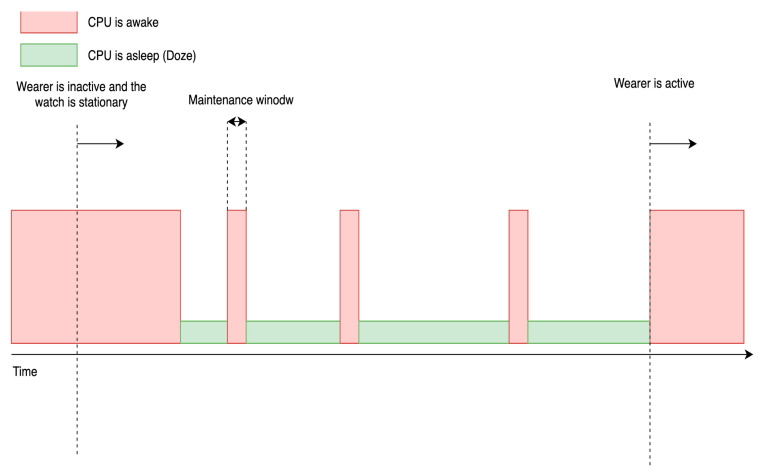
Illustration of doze mode implementation of Wear OS. The red section is when the CPU wakes up and runs all the pending tasks. The green section is when the CPU is put to sleep and ignores all tasks. The interval between the maintenance windows gets longer until the user interacts with the device again.

Upon obtaining the necessary permissions, the data collection application must prepare a service to handle background monitoring tasks. One notable challenge in this regard is the implementation of the Wear OS doze mode, which restricts long-running background tasks. Doze mode is activated when users have not interacted with the device for an extended period. During doze mode, the CPU is put to sleep to conserve battery, and certain restrictions apply. Pending background tasks are deferred to a brief maintenance window when the CPU briefly wakes up to execute them before returning to doze mode. The interval between maintenance windows increases as device idle time lengthens until the user interacts with the device again. Figure 4 depicts the operation of the doze mode maintenance window. Additionally, all alarms used to trigger background tasks are also deferred to the maintenance window. This deferral of tasks can pose challenges for time-sensitive background tasks, such as collecting sensor data at fixed intervals. In Section 5, we elucidate methods to bypass these challenges.

**Figure A2:**
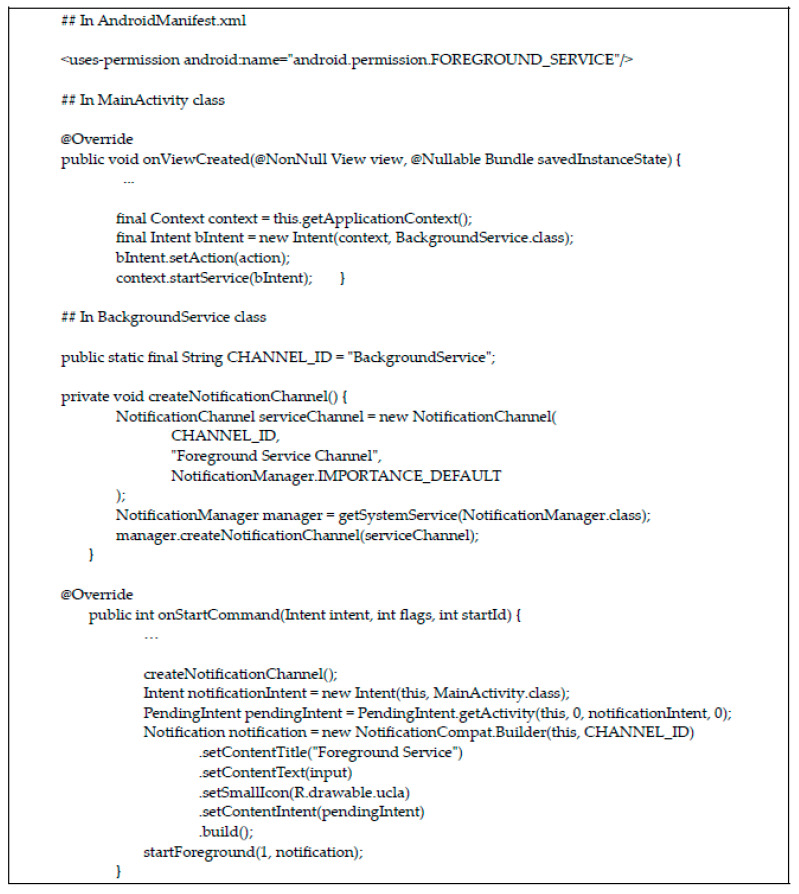
Example Android studio java code to setup foreground service.

To ensure the continuity of background tasks during doze mode, a foreground service must be employed. This Android service class incorporates notification bars to inform users about the service running in the background. By executing tasks through a foreground service, the imposed restrictions of doze mode can be bypassed, rendering it a powerful tool. Sample code for setting up the foreground service can be found in Appendix A Figure A2. However, it is important to note that utilizing this method will activate the CPU to perform background tasks, resulting in a potential impact on the battery life of the smartwatches. Subsequent sections provide more comprehensive details on battery life conservation strategies.

### 4.2. Signal Class

Healthcare monitoring tasks can be categorized into two main types: asynchronous recurring tasks, such as collecting sensor data at predetermined intervals, and sequential tasks, which involve uploading data to a remote service, requiring Internet availability checks, authentication, data transmission, and confirmation from the server. To ensure the orderly execution of these tasks, a reliable signal delivery system is crucial. Wear OS offers three systems—BroadcastReceiver, AlarmManager, and LiveData—that can be combined to serve as the signal delivery system for both types of tasks [28,29,30,31].

**Figure A3:**
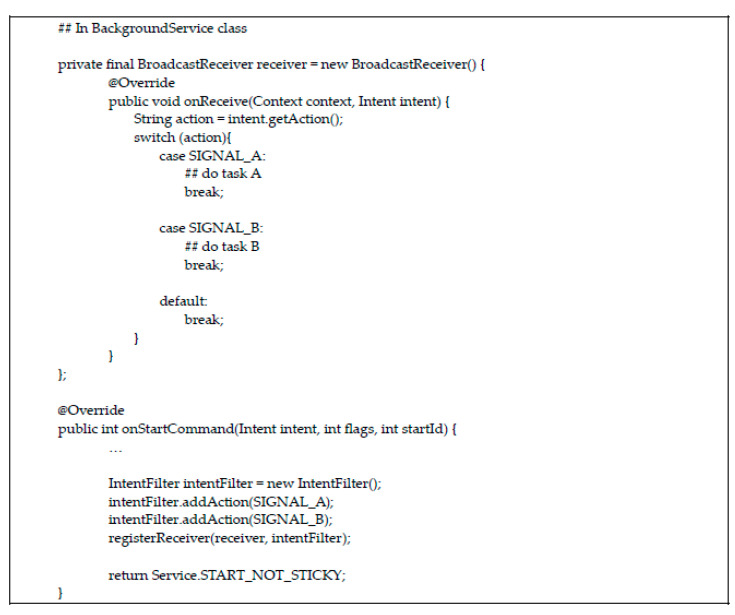
Example Android studio java code to setup broadcast receiver.

The BroadcastReceiver serves as the primary signal mechanism for general applications [28]. It operates in the background, awaiting registered triggering signals to wake up and execute tasks accordingly. For instance, a BroadcastReceiver can be registered with signals such as STEP_COUNT and PROXIMITY_SCAN. Upon receiving the STEP_COUNT signal, the BroadcastReceiver collects pedometer data, while the PROXIMITY_SCAN signal prompts it to scan for nearby proximity sensors. The triggering signals, known as intents, are registered with the BroadcastReceiver during its creation, allowing any modules within the same application to send out the triggering signals. Appendix A Figure A3 presents a sample code demonstrating the setup of a BroadcastReceiver in Android.

**Figure A4:**
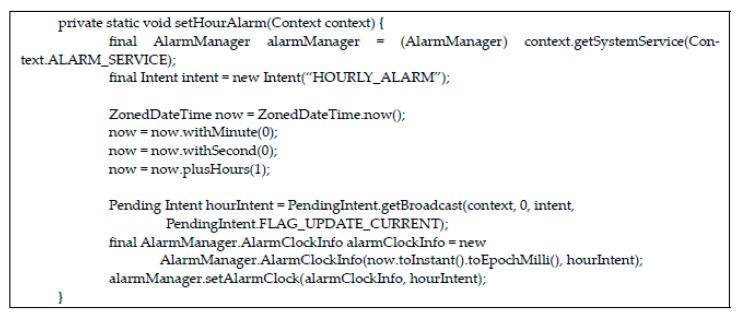
Example Android studio java code to setup alarm to go off at the start of the next hour.

The AlarmManager, often used in conjunction with the broadcast receiver, sends out triggering signals at specific times or intervals [29]. Developers can specify the exact time for the alarm to fire and determine whether it will repeatedly fire after a certain interval. This alarm system is particularly useful for tasks that require regular scheduling or precise timing, such as conducting a survey every Thursday at 5 p.m. It is important to note that during doze mode, only three functions—setAndAllowWhileIdle, setExactAndAllowWhileIdle, and setAlarmClock—are allowed to run in the background. For continuous background monitoring tasks, we recommend utilizing these three functions to avoid the signal being deferred by the operating system. Appendix A Figure A4 provides a sample code illustrating the setup of an alarm in Android.

LiveData, on the other hand, triggers tasks when associated values change and is primarily used to update UI components [30,31]. For example, a LiveData task can be associated with the “step_count” value, and whenever there is a change in the “step_count” value, the LiveData task will run to update the UI with the latest value. The advantage of this type of signal is that it relieves the UI from constantly checking for new values in the background while ensuring real-time updates. Further details on setting up LiveData for UI components are discussed in the following section.

### 4.3. Sensor Class and Data Collection Architecture

Smartwatch applications can collect user data through two types of components: passive sensors (accelerometer, gyroscope) and active sensors (Wi-Fi/Bluetooth/GPS and speaker/recorder). Passive sensors operate independently from the CPU, allowing them to function even when the CPU is in low-power doze mode. The CPU periodically wakes up to read the data stored in the sensors’ buffer. On the other hand, active sensors require the CPU to be in full-power mode every time they are controlled, making them the most power-consuming components of the smartwatch. Considering these factors, it is important to focus on the configurations and settings that enhance battery efficiency while describing the sensors used in our system.

#### 4.3.1. Passive Sensors

Passive hardware sensors capture raw data directly from the device’s modules and chips, including components like the accelerometer and gyroscope. On the other hand, software sensors utilize the built-in functions of the Operating System to process the raw data obtained from the hardware sensors to infer specific data types. An example of this is the calculation of a user’s pedometer, which is derived from the data collected by the accelerometer and gyroscope.

The sensors’ data are characterized by three primary attributes: timestamp, sensors’ measurement, and accuracy. The timestamp represents the time in nanoseconds corresponding to the sampling rate. Sensors’ measurement refers to the floating-point value reading obtained. Accuracy denotes the reliability of the sensors’ reading, ranging from 0 to 3, with 3 being the most dependable. For instance, the accuracy of heart rate reading is assigned a value of 3 when the operating system detects that the watches are tightly worn and 0 when the watch is not worn at all.

Each sensor within the smartwatch encompasses three configuration types: (a) sampling interval, (b) report interval, and (c) wake-up/non-wake-up. The sampling interval determines the frequency at which sample data are transferred to the sensors’ buffer storage, where the data are temporarily held until the CPU reads them. The report interval determines how often the CPU reads all the data present in the sensors’ buffer. The wake-up/non-wake-up configuration determines whether the CPU is compelled to awaken and read the data when the sensors’ buffer becomes full, regardless of the report rate.

The configuration of sensors has a significant impact on battery consumption and buffer capacity, thereby influencing the overall performance of the data collection application. Careful consideration should be given by programmers when determining these parameters. Often, there exists a tradeoff between optimal battery consumption and the required sensor performance, with the most suitable settings determined empirically.

Frequent wake-ups of the CPU due to shorter report intervals lead to increased power consumption. Thus, the general strategy should be to maximize the report interval as much as possible. The maximum report interval, without any loss of data, is calculated by dividing the buffer capacity by the sampling frequency (Equation (1)).

Optimal report interval = buffer capacity/sampling frequency(1)

An additional complexity arises due to the presence of two types of buffers, i.e., reserved and shared. The reserved buffer is dedicated to specific sensors, such as the accelerometer/gyroscope sensor, wherein an independent buffer storage is employed. The shared data buffer on the other hand caters to other sensors, allowing for their concurrent utilization. The reporting interval, as determined by Equation (1), facilitates the CPU’s activation and subsequent data retrieval from all sensors before the buffer attains its maximum capacity. It is imperative to recognize that not all sensors are equipped with buffers. For instance, sensors like the pedometer or worn-status sensor merely retain the most recent value. Consequently, to avert data loss, these sensors consistently awaken the CPU whenever a new value is updated. The list of sensors with buffer types and power consumption available on Android smartwatches is shown in Appendix A Table A1. We should note that companies are investing efforts in designing chipsets tailored for wearables to optimize battery consumption and overall device performance. Examples include Qualcomm’s Snapdragon series and Samsung’s Exynos series, which focus on specialized chipsets, leading to improved battery efficiency and performance in wearable devices.

##### Sensors with Buffer

This category encompasses various types of sensors, including accelerometer, gyroscope, magnetometer, barometer, linear acceleration (excluding gravity), and gravity. These sensors are characterized by their buffer capacity, which permits the Central Processing Unit (CPU) to adopt a low-power mode during report intervals, thereby optimizing battery longevity. A sampling rate of 20 Hz has been validated as effective for facilitating the day-long operation of these sensors, ensuring precise activity recognition [32]. For sustained background functionality, the data collection application aligns the sensor’s report interval with the designated buffer capacity. An exception is noted for the gyroscope sensor, attributed to its elevated power consumption relative to other sensors in this category. The gyroscope sensor is selectively employed to facilitate the classification of intricate activities beyond the capacity of accelerometer data alone [33]. Within the data collection application, the report rate for the accelerometer is established at 10 s intervals, and the data procured are directed towards an activity classifier to ascertain the user’s ambulatory status. This walking state classification (walking/nonwalking) then serves as a basis for enhancing the power efficiency of Bluetooth localization.

##### Sensors without Buffer

Heart rate, ambient light, orientation, wear state, wrist tilt gesture, geometric rotation vector, step counter, step detector, and significant motion sensors are among the sensors without buffer. For instance, the data collection application selectively employs the heart-rate, step counter, wear state, and ambient light sensors. Given their absence of buffer capacity, immediate data retrieval is essential upon availability to avoid loss. This necessitates a concurrent sampling and report interval or necessitates operation in wake-up mode. Given the Android operating system’s configuration to retrieve all sensor data upon activation, the sensors in this category are programmed with sampling and report intervals of 10 s. This synchronicity permits the operating system to conduct simultaneous data reading from both sensor types [6,7].

#### 4.3.2. Active Sensors

As previously stated, active sensors are characterized by their substantial power consumption due to the continuous engagement and maintenance of the Central Processing Unit (CPU) in a wakeful state. Consequently, the predominant strategy to mitigate power usage for these sensors is to employ them in short, concentrated bursts, thereby employing a high sampling rate within condensed timeframes.

##### Bluetooth

Bluetooth sensors, notable for their extensive utility, are incorporated in the data collection application in conjunction with proximity beacons to determine indoor patient localization. Proximity beacons propagate their presence to Bluetooth-enabled devices, a feature harnessed by our SARP system to calculate the proximity of smartwatches to beacons using Received Signal Strength Indicator (RSSI) values, thus deriving patient indoor locations. The utilization of Bluetooth Low-Energy (BLE) beacons for contextual awareness collection has gained momentum due to their durability and cost-effectiveness. Like lighthouses, these beacons emit signals broadcasting their presence that can be perceived and interpreted by Bluetooth modules embedded in wearable devices, thereby enabling the indoor location of the wearable device to be calculated. Broadcasting at a power of −80 dBm and 250 ms intervals enables these beacons to cover a 6 m radius. More extensive analyses of Bluetooth beacons’ radiation emission pattern can be found in [15,34,35,36,37].

Given the substantial power consumption of the Bluetooth antenna, it is advised to confine scanning for proximity Bluetooth beacons to periods when the accelerometer detects user movement. Indoor location is ascertained in the data collection application by considering the top three beacons with the highest mean RSSI power received by the smartwatch’s Bluetooth module. Nevertheless, this location estimation method may suffer accuracy deficits due to increased environmental objects. As such, rather than calculating the indoor localization on the wearable itself, exhaustive scan results are documented and transmitted to a cloud server, which will apply intricate analysis techniques, such as fingerprinting and triangulation, yielding more precise location tracking.

**Figure 5:**
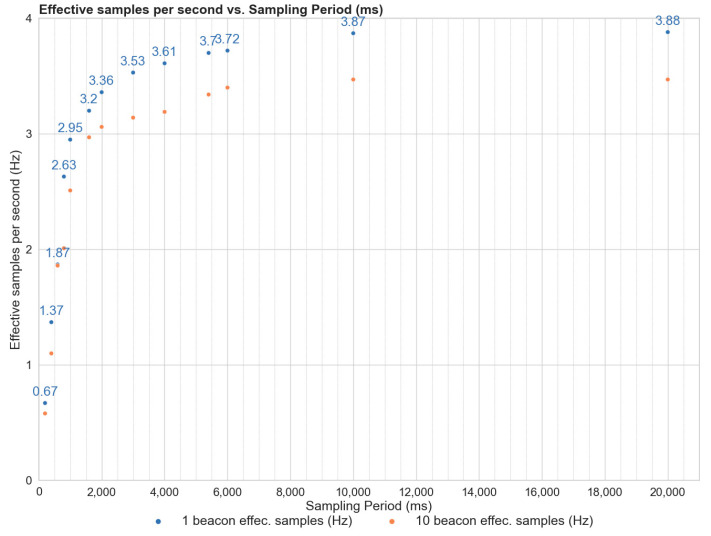
Detection characteristic of a smartwatch upon different number of beacons.

An evaluation of the smartwatch Bluetooth module’s behavior when scanning proximity beacons was conducted to better comprehend beacon behavior and assess battery consumption under differing beacon configurations. A smartwatch equipped with a 2.4 GHz Bluetooth 4.1 BLE + BR/EDR module was positioned centrally in an unoccupied room, facing upward. For each test case, a single beacon was arranged within a one-meter radius of the smartwatches, and the experiment was repeated with ten beacons within the same radius. Scanning intervals for the beacons varied from 0.25 to 10 s, with each scan recording the number of beacon detections. The results, displayed in Figure 5, suggest a stabilization of the detection rate at approximately three detections per second after two seconds. An increase in beacon quantity slightly decreased this rate. Therefore, we calibrated the scanning interval to ensure sufficient detection counts for indoor localization calculation. Considering that beacon signals are prone to interference from the environment, the orientation of smartwatches, and the human body, we determined 3.6 s to be the optimal scanning interval to balance battery preservation with data collection for user location tracking [15,35,37].

The pervasive proliferation of Bluetooth technology has enabled a variety of health monitoring devices to transmit their readings through Bluetooth, thereby making smartwatches an emergent central hub within the ecosystem of home health devices. This mode of communication mirrors the prevalent adoption of the Generic Attributes (GATT) data structure system across Bluetooth-enabled devices, enhancing interoperability. These devices, employing distinct Universally Unique Identifiers (UUID) [38], broadcast their available services and data in accordance with the GATT standard [23]. Devices intending to access these data can establish a pairing with the broadcasting devices and subscribe to the available services and data via the UUID. It should be noted that not all Bluetooth health devices currently on the market rigorously adhere to this process (for example, iHealth and Buerer’s devices employ custom service protocols for data reading) [39,40]. Nevertheless, for the devices that do comply with this standard (such as Polar, A&D, and Contour Next) [41,42,43], the procedure for data reading remains relatively straightforward.

##### Wi-Fi/4G

The utilization of Wi-Fi networks constitutes an additional method for indoor localization, primarily due to three factors: (1) the proliferation of smartphones equipped with GPS and Wi-Fi, (2) the prevalent practice of users leaving their device’s Wi-Fi and GPS enabled, and (3) the periodic background transmission of status reports, inclusive of nearby routers’ IP and GPS location, by Android devices to Google. Consequently, in the absence of Bluetooth beacons, Wi-Fi provides a swift and efficient approach to ascertain a user’s general location (for example, the building they occupy). By default, the Android operating system permits applications to perform scans once every half hour. This frequency does not impart a discernible impact on the battery life of smartwatches.

The primary function of the Wi-Fi antenna in the data collection application is to facilitate the synchronization of data with the remote cloud server. This synchronization process involves transmitting sensor data and device status to the IoT-Hub, interpreting remote configurations, and updating the watch time. With consistent access to a Wi-Fi network with an average upload speed of 20 Mbps, our data collection application only requires a few minutes of Wi-Fi usage per hour for smartwatches to successfully complete all cloud synchronization tasks. This includes uploading less than 3 MB of sensor data per hour, considering activities such as accelerometer sampling at 25 Hz, Bluetooth scanning during user movement, heart rate sampling every minute, encoded survey responses, and more. Some of the files in the synchronization task may remain pending due to Wi-Fi issues or watches running out of battery. Then, the data collection application will resume the leftover cloud synchronization tasks while the user charges the smartwatch, as long as a Wi-Fi network is available. This guarantees that user data remains current and updated. Additionally, the Wi-Fi antenna can be leveraged to extend its functionality by employing geolocation techniques, utilizing the wearer’s current IP address, for the purpose of tracking the wearer’s location.

##### GPS

Among all antennas, the GPS antenna demonstrates the highest power consumption per reading. The requirement for maintaining connections to weak signals from multiple satellites for durations between 10 to 60 s to yield a single reading necessitates considerable power. Consequently, the activation of this antenna should be reserved for situations where alternative localization methods described earlier are unfeasible or during emergency alerts. To ensure that the smartwatch can function for an entire day with all other sensors and antennas being operational, the recommended interval between GPS scans should be longer than 10 min [44].

##### Speaker/Recorder

The speaker and recorder functionalities of smartwatches are employed to facilitate a weekly survey within the data collection application. Given the compact screen size of smartwatches, the built-in voice recognition feature provided by Wear OS is optimally used to capture users’ responses. The smartwatch’s speaker vocalizes the survey questions while simultaneously presenting it on the screen. Users have the choice to respond either by selecting an answer on the screen or through voice command. An adaptive survey approach can also be utilized, where subsequent questions are determined by the remote server based on users’ preceding responses. Upon completion of the survey, the smartwatch documents the responses and uploads them to the remote server for storage. The surveys can be scheduled to run at customizable time intervals (e.g., hourly, daily, or monthly). The survey content should be designed to allow the user to complete it quickly to minimize the time the CPU needs to be awake and conserve the watch’s battery.

##### Sensors Setup for Activity and Indoor and Outdoor Location Tracking

**Figure 6:**
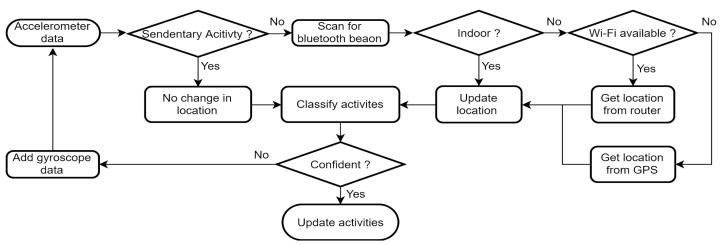
Decision rule to track users’ activity and location using the combination of accelerometer, gyroscope, Bluetooth, Wi-Fi, and GPS sensors while minimizing power consumption.

By integrating a range of sensors, including accelerometers, gyroscopes, Bluetooth, Wi-Fi, and GPS, a comprehensive tracking system can efficiently monitor users’ activities both indoors and outdoors, which can help depict a comprehensive storyline of patients’ daily routines and behaviors. In our application, the accelerometer is utilized to continuously capture data in the background, determining whether the wearer is in motion or stationary using a pretrained classifier embedded on the watch. When the user is stationary, the data collection application conserves battery power by excluding the reading of location-related sensors, focusing solely on the accelerometer to identify potential changes in physical activity [13]. In instances where the classification result lacks confidence, supplementary data from the gyroscope sensor is collected to enhance the accuracy of the classification. When the user is in motion, the application updates the location by employing the Bluetooth sensor to scan for nearby proximity beacons. If beacons are detected, the indoor location is determined based on the strongest RSSI; otherwise, it is assumed that the user is outside their residence. In such scenarios, the smartwatch checks for the availability of Wi-Fi to determine the location based on the IP address or resorts to GPS if Wi-Fi is not accessible. Figure 6 illustrates the decision tree rule employed by the data collection application for activity and location tracking, taking into consideration battery consumption concerns at each stage.

### 4.4. User Interface (UI) Class

In the interest of conserving battery life, we recommend employing a black background within the user interface. By limiting the illuminated portion of the screen to merely a fraction of the total available pixels, the associated energy expenditure becomes negligible. Moreover, the brightness level of the screen is strategically reduced whenever feasible, leveraging data from the ambient light sensor. In instances of user interaction, the screen can temporarily be brightened for a few seconds.

Another method to save battery is by utilizing the LiveData class for the UI thread, as described in Section 4.2. Typically, the CPU needs to be fully awake at regular intervals to run the UI thread and check for any updates to UI elements (e.g., watch clock time or step counter). With LiveData, any changes in the UI element are buffered and updated later when the CPU is fully active. This approach is particularly beneficial when the user does not interact with the watch for an extended period, as the watch enters doze mode to save battery life. LiveData allows the app to defer UI changes until the CPU wakes up again, either during a periodic maintenance window or when the user interacts with the watch, thereby prolonging battery conservation mode.

### 4.5. Network and Monitoring Class

In our case study, both the administration and the data collection applications consistently record their activities into designated log files. Each activity log is diligently timestamped on a daily basis, encompassing the time, day, month, and year. These log files are securely retained in storage until they are scheduled for upload to the cloud. The essence of these logs is paramount for conducting security audits and facilitating developmental processes.

At the top of each hour, the data collection application attempts to transmit a series of information to the IoT-Hub. This information includes sensor data, the latest version of the log files, and pertinent device status information, such as battery level and the most recent charging instance. When the smartwatches find themselves in a charging state, the administration application initiates a retrieval of remote configuration data from the IoT-Hub. These configurations encompass a spectrum of modifications, from minor adjustments like survey details, UI language, and survey initiation timings to more substantial updates, such as application upgrades for both the administration and data collection applications. Following the successful execution of these configurations, the administration application cedes control to the data collection application, allowing it to execute its routine activities for the subsequent hour.

### 4.6. Data Collection Application Deployment through Administration Application

In a conventional Android application deployment scenario, developers submit their applications to the Android app store, where they undergo scrutiny for potential malicious activity. After successfully navigating this review process, the applications become accessible on the Android app store, ready for download via the Google Play application on users’ devices. This standard model, however, can prove unsuitable for healthcare monitoring applications akin to ours, which primarily targets the geriatric population for remote patient monitoring. The reasons for this incongruity may include the following:

1. The Google Play application requires the presence of Google accounts on smartwatches. Given that our primary users constitute the geriatric population, many of whom may be less familiar with smart device technology, reliance on the Google Play application could impose unnecessary complications. While it is feasible to create Google accounts for each user, this strategy could inadvertently introduce additional security vulnerabilities into the system.
2. The security of users’ personal data can be compromised when the application is made available on the Android store. Potential malicious actors could download the applications and employ reverse engineering tools, such as Apktool, to identify any hardcoded credentials within the application [45].

One plausible solution to circumvent these issues involves direct installation of the data collection application onto devices via console command, bypassing the Android app store. However, this approach would impede our ability to update the data collection application, given that typical Android applications are restricted from installing other applications absent from the Android app store. Furthermore, console command usage is a less accessible approach, especially for our target demographic—the geriatric community.

Beyond the complexities of application deployment, the data collection application faces potential issues related to storing user credentials on devices. Every Android device has a debugging mode, enabling the execution of custom shell commands. There exists a particular command of concern that roots devices, thereby granting potential hackers access to sensitive data and credentials. Furthermore, it opens the possibility for the installation of malicious software, thereby compromising both the devices and any connected cloud systems. This factor poses a considerable issue for any application, particularly ones similar to ours which necessitate long-term storage of user credentials on devices in an effort to minimize user interaction.

Our framework introduces an ancillary administration application on smartwatches to address these security concerns. This application operates in “Device Owner mode” [46], which facilitates a level of direct control over the devices not attainable by a typical Android application. The salient features of this mode, as they pertain to our application, include the ability to disable debugging mode and the capability to programmatically install custom applications.

The disabling of debugging mode: This action serves to thwart any efforts to execute shell commands on the devices, thereby preventing potential hackers from gaining root access to the devices to steal security information or install malicious applications. The only way to re-enable the debugging mode on the devices is via a factory reset, which concurrently erases the administration application and sensitive data during the process. This approach safeguards the users’ sensitive data and credentials stored on the devices by precluding the possibility of device rooting.

**Figure 7:**
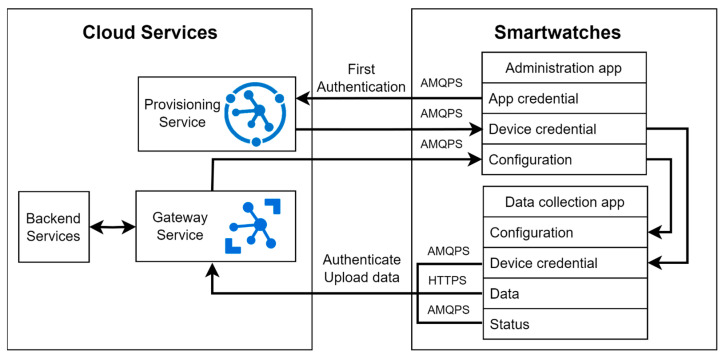
The complete network communications between the administration app, the data collection app, and the remote server for the data collection app deployment, data uploading, and remote monitoring.

The installation of custom applications programmatically: This feature permits direct oversight of the data collection application distribution, as it no longer relies on the Android store. Instead, we utilize the Azure Device Provisioning Service (DPS) [47] in conjunction with the Azure IoT Hub Service (IoT-Hub) for the data collection application distribution. During the initial setup, the administration application authenticates itself with the DPS. The DPS, in turn, registers a device ID on the IoT-Hub and returns that ID’s credentials to the administration application. Subsequently, the administration application downloads the actual data collection application via the IoT-Hub and initiates its installation. This method provides the added advantage of enabling the IoT-Hub to monitor and remotely configure the data collection application, as described in the previous networking and monitoring class section. Figure 7 illustrates the complete network communications between the administration app, the data collection app, and the remote server for the data collection app deployment, data uploading, and remote monitoring.

## 5. Notes to Consider

### 5.1. Standalone or Smartphone-Dependent

Applications crafted for Wear-OS-enabled smartwatches predominantly adhere to a design philosophy in which each application is envisaged as a companion to a smartphone to execute an action. Given the tendency of our target audience to either not possess smartphones or to lack advanced technological literacy, the necessity to develop standalone applications becomes paramount, effectively eliminating the requirement for smartphones. This approach presents an added advantage of mitigating potential user frustration arising from technical complications. However, it should be noted that several sections of this paper, such as those discussing the data collection application deployment through administration applications, are aimed at addressing challenges predominantly arising from developing a standalone application that operates without a smartphone. 

A further challenge posed by standalone applications pertains to the mandatory initial watch pairing setup imposed by all smartwatch manufacturers, which necessitates pairing the watch with a phone. To circumvent this restriction for the deployment of remote patient monitoring applications, an initial setup procedure is employed. During this setup, smartwatches are temporarily paired with a smartphone to satisfy the pairing prerequisite. Once this prerequisite is met, the smartphone Bluetooth connection can be disconnected, which eliminates the pairing data, thereby facilitating the independent operation of the smartwatches without the persistent necessity for a paired phone.

### 5.2. Background Execution Limit

Most smartwatch OS platforms have runtime restriction policies to save battery consumption that developers need to follow to have their applications made available on the app store of the platform. The most disruptive one is the background execution limit, which constrains the amount of time the application can run in the background before being put to sleep by the OS. However, our application needs to run continuously in the background without interruption. Under the background execution limit, after 15–20 min of no hand movement, the smartwatches will enter a sleep state to conserve battery. The smartwatches will only wake up their CPUs for a minute in 10 min intervals for the app to collect sensors’ data. This disruption will cause significant data loss during the deep sleep period. Currently, there is no option for Wear OS smartwatches to whitelist the app from this limit. To solve this issue, according to Google guidelines [48], our app will need to register a notification channel for the app. This will help the OS to consider the app as active and will not put the app to sleep when the wearer is inactive.

Most smartwatch operating system (OS) platforms enforce runtime restriction policies to mitigate battery consumption, a prerequisite that developers must abide by for their applications to be listed on the platform’s app store. Among these constraints, the background execution limit, which curtails the duration an application can operate in the background before the OS places it into a dormant state, is particularly intrusive. However, remote patient monitoring applications normally necessitate uninterrupted, continuous operation in the background. Given the background execution limit, the smartwatches will transition into a power-conserving sleep state after approximately 15–20 min of no hand movement. During this deep sleep period, the smartwatch’s CPU will only awaken at intervals of ten minutes for a single minute, permitting the data collection application to gather sensor data. This interruption leads to significant data loss during the deep sleep period. As of the time of this manuscript’s preparation, Wear OS smartwatches offer no provision to exempt an application from this constraint. To address this predicament, the data collection application, as per Google guidelines, will need to register a notification channel. This approach will enable the OS to perceive the data collection application as active and forestall the induction of a sleep state during wearer inactivity [46].

### 5.3. Handling Time without Paired Smartphone

Wear OS inherently ensures the synchronization of the smartwatches’ date and time via either a Wi-Fi network or paired phone. Nonetheless, the time zone on the smartwatch can be synchronized exclusively through a paired phone or user intervention. In the data collection application, we implemented time synchronization utilizing an API from ip-api.com, accessed on 17 July 2021. This API enables the retrieval of the time zone from the users’ Wi-Fi router, and the data collection application synchronizes its time accordingly. In the case of Wear OS version 2.2 (released in November 2018) with a target application level of 28, the administration application we described earlier can request the OS to modify the system time zone as needed. However, for applications older than this version, Wear OS currently does not provide an option to alter the system time zone using an administration application. Consequently, users are required to manually adjust the system time zone upon traveling.

### 5.4. Control Wi-Fi Switch

In a typical Android application, the process of synchronizing data with Google servers consumes approximately 2–3 h of battery power when Wi-Fi connectivity is available. To optimize battery life, smartwatches are designed to keep the Wi-Fi functionality disabled, except during designated synchronization periods with cloud services. However, in situations where the application requests the operating system (OS) to disable Wi-Fi and the watch is not actively paired with a nearby phone, the OS system briefly disables Wi-Fi before reactivating it. To address this issue, immediate action can be taken upon the installation of the administration application on the smartwatches. By granting the “android.permission.WRITE_SECURE_SETTINGS” permission to the administration application via a console command, the application gains the capability to activate airplane mode. Notably, the Bluetooth module, which is crucial for indoor localization within our application, is excluded from airplane mode. It is important to emphasize that this permission is granted only once, following the installation of the administration application, and cannot be modified subsequently, as the ability to execute console commands is no longer available. Furthermore, it is noteworthy that this privilege does not revert, even after installing system updates.

### 5.5. File Write

**Figure A5:**
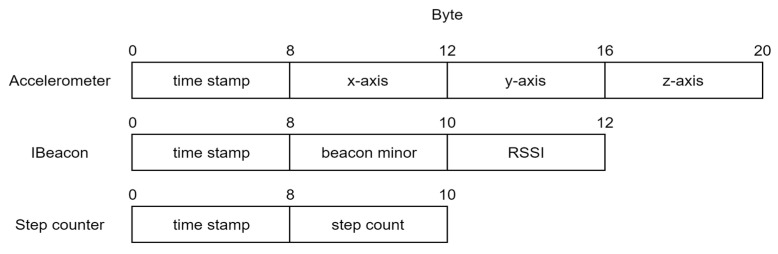
Sample binary file format to save disk space.

The data produced by the data collection application are stored in a private directory, which is allocated by the operating system for enhanced security. By defining a custom binary storage format rather than traditional file formats, such as CSV, XML, or JSON, it is feasible to drastically reduce the size of the files. In the data collection application, the binary format for various data types, shown in Appendix A Figure A5, amounts to merely 1/5th of the size of its CSV-formatted counterpart. For instance, an average 1 h data file, which contains accelerometer data sampled at a frequency of 20 Hz, approximates to around 1 MB. The remainder of the collected data, in comparison with accelerometer data, generates less than 1 kB of data per hour, making its size relatively negligible. Given the 2 GB of storage provided by the smartwatch, it can hold up to two months’ worth of data in the absence of Internet connectivity for transmission to cloud storage.

### 5.6. Dual Communication between the Administrative App and Patient Monitoring App

The data collection application transmits its operational status to our administration application at one-minute intervals. This communication proves to be integral for the application’s consistent functioning, given the imperative nature of coordinating data transmission and software updates to circumvent potential data losses. For instance, a scenario where the administration application initiates an update to the data collection application while the latter is in the process of data transmission could potentially result in data loss. Additionally, in an effort to conserve memory space, Wear OS occasionally terminates running applications. Given that our data collection application is continuously operational, it runs the risk of being terminated by the OS in the event of low available RAM on the smartwatch. In such instances, the administration application detects the termination and proceeds to restart the data collection application. This functionality also proves useful in the event of unexpected crashes; by restarting the data collection application, crash reports can be sent to the remote server, thereby aiding in debugging efforts.

## 6. Limitations of Smartwatch Applications and Future Research

In this section, we delineate the limitations encountered by developers when constructing remote patient monitoring applications that necessitate continuous operation on smartwatch platforms. It is important to note that while our primary focus is on Android Wear OS, we acknowledge that other platforms impose comparable constraints on developers.

Reliance on the Wear OS platform presents a significant limitation for smartwatch-based healthcare applications. The dynamic nature of the Wear OS platform can introduce uncertainties over time, particularly affecting the uninterrupted operation of remote monitoring applications. Wear OS is designed to restrict background applications in order to optimize battery consumption, thereby conflicting with the requirements of these applications. A notable constraint is the background execution limit described in Section 5.2. Before API level 26, Wear OS allowed applications to run continuously in the background without any time limitations. However, this behavior changed with the introduction of API level 26, as background applications are paused when the watch enters doze mode, resulting in the cessation of sensor data collection. To address this concern, we propose the utilization of a foreground service to ensure that sensor operations persist even during doze mode. Despite the fact that the most recent API level 30 has not modified this behavior, it cannot be guaranteed that future versions of Wear OS will retain the same characteristics, potentially necessitating substantial adaptations to accommodate the new API.

Another limitation of the Wear OS platform pertains to the absence of mechanisms for distributing applications without mandating a Google account registration on the smartwatch. Google Play serves as the primary channel for application distribution and updates within the Wear OS ecosystem, necessitating a Google account for these purposes. In our proposed remote patient monitoring (RPM) system, we leverage the “DeviceOwner” functionalities of the smartwatch to establish an administrative application responsible for application distribution, bypassing the reliance on Google Play. However, it is important to note that not all Wear OS smartwatches support this particular feature. For instance, the Samsung Galaxy Watch 5, a recent model, encompasses this feature, whereas the TicWatch Pro 3 GPS does not. Therefore, it is imperative for developers to ascertain the availability of DeviceOwner capabilities on their targeted smartwatch prior to determining the suitability of our proposed method.

A standardized benchmark for ensuring the consistent quality and behavior of remote monitoring health applications on Wear OS platforms is currently lacking. However, developers can leverage native tools available on the platform, namely Android Profiler, Microbenchmark, and Macrobenchmark, to address this issue. Android Profiler facilitates the measurement of application performance in terms of CPU utilization, memory consumption, and power consumption. Microbenchmark empowers developers to assess specific components of the application that are frequently executed, ensuring they meet predefined target metrics related to CPU, memory, network, power, GPU, network, and machine learning. Similarly, Macrobenchmark enables the evaluation of overall application performance during typical user interactions. In our forthcoming research endeavors, we intend to integrate these three tools to establish comprehensive guidelines for quality assurance in remote monitoring health applications on smartwatches. 

Developers should be aware that emerging sensor technologies, such as flexible and skin sensors, are currently under investigation to be integrated into healthcare ecosystems. It is essential to conduct further research to assess the efficacy of incorporating these sensors alongside wearable technologies. Such investigations are critical to inform decision making and optimize the potential benefits in healthcare applications.

## 7. Conclusions

This paper tried to demonstrate the potential of using smartwatches as a means of continuous patient monitoring in healthcare. The popularity of wearable devices, particularly smartwatches, has provided an opportunity for healthcare and telemedicine providers to leverage these technologies for various purposes, such as reducing hospitalization costs, improving personalized health and care management, and monitoring patients in remote and rural areas. While smartwatches offer promising capabilities for patient monitoring, their adoption in healthcare is still in its early stages. Existing research primarily focuses on feasibility studies, and there is a lack of comprehensive investigations into the challenges associated with developing smartwatch-based applications for continuous healthcare data collection.

This paper presented a guidance to design an Android smartwatch app for remote monitoring of geriatric patients. We demonstrated several obstacles in app development, including short battery life, wearable comfort, subject compliance, operating system app termination behavior, difficulty in user interaction due to small touch screens, personalized sensor configuration, and the ability to connect to other monitoring devices. By shedding light on these challenges and providing insights into the design decisions and implementation details of the smartwatch app, this paper aims to assist other programmers and healthcare professionals in developing more efficient healthcare applications. The findings and experiences shared in this study can serve as valuable guidelines and lessons learned for future endeavors in smartwatch-based patient monitoring.

As the field of wearable technology continues to evolve, it is crucial for researchers, developers, and healthcare practitioners to address the challenges associated with smartwatch-based healthcare applications. With further advancements and innovations, smartwatches have the potential to revolutionize patient monitoring, enhance healthcare outcomes, and bridge the gap in access to care, particularly in remote and underserved areas. By overcoming the identified obstacles and leveraging the capabilities of smartwatches, we can pave the way for a more patient-centric and efficient healthcare system.

## 8. Patents

The Sensing At-Risk Population system is protected by a patent (US patent 10937547) [15] owned by the University of California, Los Angeles, in which RR, AE, and AN are listed as co-inventors. 

## Figures and Tables

**Table 1:** List of relevant tasks requiring permission with their corresponding permission name.

Task	Required Permission
Using Internet	android.permission.INTERNET
Check network connection state	android.permission.ACCESS_NETWORK_STATE
Using Bluetooth to discover and pair device	android.permission.ACCESS_FINE_LOCATION
	android.permission.BLUETOOTH_ADMIN
Using onboard sensor such as accelerometer, heartrate	android.permission.BODY_SENSORS
Detect when the watch restarts	android.permission.RECEIVE_BOOT_COMPLETED
Using audio recorder	android.permission.RECORD_AUDIO
Wake up the CPU from sleep	android.permission.WAKE_LOCK
Read and write data to external storage	android.permission.READ_EXTERNAL_STORAGE
	android.permission.WRITE_EXTERNAL_STORAGE
Use vibration to notify wearer	android.permission.VIBRATE
Retrieve device serial number	android.permission.READ_PHONE_STATE

## Data Availability

No new data were created.

## Appendix A

**Table A1:** Details of all sensors on the smartwatch used in the data collection application.

Sensor	Sensor Type	Reserved Buffer (byte)	Shared Buffer (byte)	Power (mA)
Accelerometer	Hardware	150	3000	0.23
Gyroscope	Hardware	150	600	6.1
Magnetometer	Hardware	150	600	0.23
Heartrate	Hardware	None	None	0.79
Ambient light sensor	Hardware	None	None	0.23
Barometer	Hardware	150	300	0.005
Orientation	Software	None	None	6.56
Wear state	Software	None	None	0.23
Wrist tilt gesture	Software	None	None	0.23
Geometric rotation vector	Software	None	None	6.56
Step counter	Software	None	None	0.23
Step detector	Software	None	None	0.23
Significant motion	Software	None	None	0.23
Linear accelerometer (excludes gravity)	Software	150	3000	0.23
Gravity	Software	150	3000	6.1
